# Modified Anatomic Locking Plate for the Treatment of Posteromedial Tibial Plateau Fractures

**DOI:** 10.1111/os.12714

**Published:** 2020-08-12

**Authors:** Zhen Jian, Rong‐guang Ao, Jian‐hua Zhou, Xin‐hua Jiang, Bao‐qing Yu

**Affiliations:** ^1^ Department of Orthopaedics, Shanghai Pudong Hospital Shanghai Fudan University Pudong Medical Center Shanghai China

**Keywords:** Anatomic locking plate, Posteromedial approach, Posteromedial tibial plateau fractures

## Abstract

**Objective:**

To evaluate the safety and clinical efficacy of a modified anatomic locking plate for the treatment of posteromedial tibial plateau fractures.

**Methods:**

A retrospective study was performed in our department. Between January 2014 and February 2017, 11 patients with posteromedial tibial plateau fractures underwent surgery with the new anatomic locking plate for the posteromedial tibial plateau *via* the posteromedial approach. The study included 7 male and 4 female patients, with a mean age at the time of the operation of 39 years. During surgery, operation time and blood loss were recorded. Clinical evaluation was performed using the Tegner–Lysholm functional score, the Rasmussen functional score, and the Rasmussen anatomical score.

**Results:**

The mean follow‐up time of the study was 35 months. The mean interval between the time of injury and the surgery was 7.4 days. Radiological fracture union was evident in all patients at 14 weeks. During surgery, the blood loss ranged from 50 to 150 mL, and the duration ranged from 55 to 90 min. The Tegner–Lysholm functional score ranged from 80 to 96 at the final follow up. Moreover, the final Rasmussen functional score ranged from 25 to 28, and the Rasmussen anatomical score ranged from 15 to 18. The mean knee arc of motion was 137° (range, 122°–153°). Symptoms of knee instability or severe pain were not found in any cases. No flexion contractures or extensor lag was seen. No infection, deep vein thrombosis, or graft site morbidity was seen at the follow up. No case of reduction loss or internal fixation failure was reported during the follow‐up.

**Conclusion:**

With the clinical data of the small‐sample‐size population (11 patients) during a 19 to 60‐month follow‐up, the modified anatomic locking plate for the posteromedial tibial plateau proved to be safe and effective and is an adequate fixation method for the treatment of posteromedial tibial plateau fractures.

## Introduction

The increase in frequency of high‐energy trauma and fragility fractures has led to more complex tibial plateau fracture patterns. They often present as a diagnostic and interventional challenge that requires early and efficient intervention to minimize long‐term complications of joint dysfunction and early osteoarthritis. Early anatomical reduction and stable internal fixation, followed by early mobilization is the principle for management of tibial plateau fractures^1^, ^2^.

Fractures that involve the posteromedial tibial plateau are often encountered after high‐energy injuries and require special attention when developing a preoperative plan^3^, ^4^. Schatzker^5^ believed fractures that occurred in the medial plateau carried the worst prognosis, likely due to the high variability and combination of bony and soft tissue injuries. Thus, recent attention on the occurrence of posteromedial fracture fragments in complex tibial plateau fractures underscores the growing appreciation for the difficulty in treating these fracture patterns^3^, ^6^. In a cadaveric model of a posteromedial tibial plateau fracture^7^, fracture fragments studied displaced with knee flexion, even at low flexion angles. Although such fragments may initially seem nondisplaced after injury, posteromedial fragments similar to these tested are likely to displace during knee range of motion exercises in non‐weight‐bearing conditions. As a result, this fragment should be considered inherently unstable^7^, ^8^, ^9^.

Some authors^6^, ^8^, ^10^ recommend a separate fixation for this fragment to maintain reduction, although lateral approaches for stabilizing medial tibial plateau fragments have had excellent early clinical outcomes^11^. They suggest that these medial‐sided fractures often require a separate medial approach and plating strategy because an isolated lateral plate will not rigidly fix an unstable medial plateau. On average, the fragment is described to encompass approximately 25% of the joint surface, with a sagittal angle around 73°–80° and around 45 mm in height^12^. The vertical fracture pattern suggests shear forces and instability that are not necessarily addressed by the current laterally based approaches to fixation. One study by Higgins *et al*.^13^ argues that dual‐plate fixation results in improved stability compared with lateral locked plating alone in bicondylar tibial plateau fractures. In a biomechanical study in composite tibiae, Yoo *et al*.^14^ compared load to failure in locked and non‐locked single and dual plating systems. The dual plating construct tolerated higher loads, with the authors hypothesizing that the lateral locking screws insufficiently reduce the posteromedial fragment.

However, the commonly used posteromedial plate is either non‐locked, and lacks angular stability, or has a proximal part that is not wide enough to wrap the entire posteromedial fragment, with contouring being difficult during surgery. Therefore, we modified a specialized anatomic locking plate for posteromedial tibial plateau fractures. The purpose of the study was: (i) to describe the details of an innovative anatomic locking plate for the posteromedial tibial plateau; (ii) to introduce the surgical method for treating posteromedial tibial plateau fractures with this modified plate; and (iii) to evaluate the safety and clinical efficacy of the innovative plate. To simplify the research, we retrospectively studied 11 patients with posteromedial tibial plateau fractures in our trauma center (lateral fixation was not needed).

## Materials and Methods

### *General Data*

The presented retrospective study was performed with the approval of our institution’s human subjects review board. Patients with a posteromedial tibial plateau fracture that underwent surgery with the anatomic locking plate for the posteromedial tibial plateau between January 2014 and February 2017 were included in our study.

The exclusion criteria were: (i) severe open fractures; (ii) multiple traumas; (iii) osteofascial compartment syndrome; (iv) pathologic fractures; (v) autoimmune diseases; (vi) blood disorders; and (vii) less than 18 months of follow‐up.

Therefore, clinical documents and radiograph images of 11 included patients with a posteromedial tibial plateau fracture were reviewed in detail. Preoperative and postoperative plain X‐rays as well as preoperative CT scans were obtained for all patients. All injuries resulted from high‐energy traumas, including battery bike‐related accidents (8 patients), falls from an elevated height (2 patients), and sport‐related injuries (1 patient). Injuries were classified into Schatzker type IV fractures and AO/OTA 41 type B fractures (partial articular). According to the CT scan and reconstruction images, the main fragments were located in the posterior half of the medial condyle and/or a fracture line impacted the posterior aspect of the medial plateau. According to the three‐column classification^2^, all cases were fractures of the posteromedial column.

Every surgery was performed after the soft tissues were stabilized. For patients who also had severe soft tissue injuries, such as tense swelling, distal bony traction and splints were applied. The mean interval between the time of injury and the surgery was 7.4 days (range, 2–12 days). The surgeries were performed by three experienced surgeons (leaded by Dr Yu) who specialized in knee surgery or trauma.

### *Surgical Technique and Rehabilitation*

Step 1: All operations were performed using spinal anesthesia or general anesthesia. The patient was placed in prone position on a radiolucent table, and the lower limb tourniquet was inflated.

Step 2: To address the posteromedial tibial plateau fracture site, a gentle S‐shaped curvilinear incision was placed on the posteromedial aspect of the knee. After sharp dissection of the subcutaneous tissue, the popliteal fascia was incised, preserving the small saphenous vein as well as the medial sural cunateous nerve. The medial head of the gastrocnemius muscle was then mobilized and retracted laterally. The oblique tendinous expansion of the semimembranosus tendon was identified and retracted medially. The superior border of the popliteal muscle was identified and the muscle was dissected subperiostally to allow for posteromedial fracture site visualization. An arthrotomy was performed to assess the reduction of the articular surface at the level of the main fracture.

Step 3: After preliminary fixation of the fracture fragment using multiple K‐wires, reduction was controlled by fluoroscopy in the frontal and lateral view. The defect was packed with artificial bone graft if needed. Step 4: At this point, the modified plate was placed to stabilize the fragment and compression was applied by placing compression screws in a posteromedial–anterolateral direction. Step 5: The quality of joint reduction, location of the plate, and length of the screws were confirmed under fluoroscopic guidance. Physical examination of the knee stability was routinely conducted to exclude ligament injury. Then, the subcutaneous tissue and skin were closed over suction drains.

Mobilization of the knee joint began immediately after surgery. In addition, a daily injection of 4100 IU low molecular heparin was applied to prevent deep vein thrombosis. All patients achieved 90° in flexion prior to discharge, usually 5–10 days after surgery. The knee range of motion, as well as muscle strength was increased immediately after surgical wound healing. Patients were initially treated as non‐weight‐bearing for 12 weeks to avoid early loss of reduction. Depending on radiographic signs of healing, partial weight‐bearing was allowed and rapidly advanced to full weight‐bearing as tolerated by the patients.

Clinical evaluation was performed using the Tegner–Lysholm functional score, the Rasmussen functional score, and the Rasmussen anatomical score. Reduction loss is an important indicator for measuring the effectiveness of internal fixation. The definition of reduction loss after tibial plateau fracture surgery is still controversial. Therefore, we used a method applied by Kim *et al*.^6^ and Weaver *et al*.^15^ Reduction loss was defined as a depression of the articular surface that is greater than 3 mm compared to the immediate postoperative radiographs, a condylar widening that is greater than 5 mm, and an alignment change that is greater than 5° towards the varus or valgus. Furthermore, reduction losses that occurred through loosening or breakage of the plate were also included.

#### *Tegner–Lysholm Functional Score*

The Tegner–Lysholm functional score is one of the most frequently used assessment tools for the functional outcomes of knee injury. Eight factors are rated to produce an overall score on a point scale of 0 to 100. The factors of limp, support, and locking are worth a potential 23 points, pain and instability 25 points each, swelling and stair climbing 10 points each, and squatting 5 points. Then an assignment is given as “excellent” for 95 to 100 points, “good” for 84 to 94 points, “fair” for 65 to 83 points, or “poor” for less than 65 points.

#### *Rasmussen Functional Score*

The Rasmussen functional score is used to evaluate knee function primarily, with special attention paid to the single plane stability and rotation stability. It consists of five categories, including pain (6 points), walking capacity (6 points), extension (6 points), total range of motion (6 points), and stability (6 points). The total score is the sum of these five items, and the higher scores indicate greater function. A score of 30 points is defined as normal, 27–30 as excellent, 20–26 as good, 10–19 as fair, and 6–10 as poor.

#### *Rasmussen Anatomical Score*

The Rasmussen anatomical score is a principal tool to evaluate reduction quality of tibial plateau fractures on radiological images after surgery. It consists of three categories, including depression (6 points), condylar widening (6 points), and angulation (6 points). A score of 18 points is defined as excellent, 12–17 as good, 6–11 as fair, and 0–5 as poor.

## Results

### *Patients*

All 11 patients underwent surgery with the anatomic locking plate for the posteromedial tibial plateau. Of the patients, 7 were male and 4 others were female. The mean age at the time of the operation was 39 years (range, 22–69 years). In addition, all patients were followed up by clinic review. The mean follow‐up period was 35 months after the surgery (range, 19–60 months). The plate details were as follows.

### *Plate Details*

**Fig 1:**
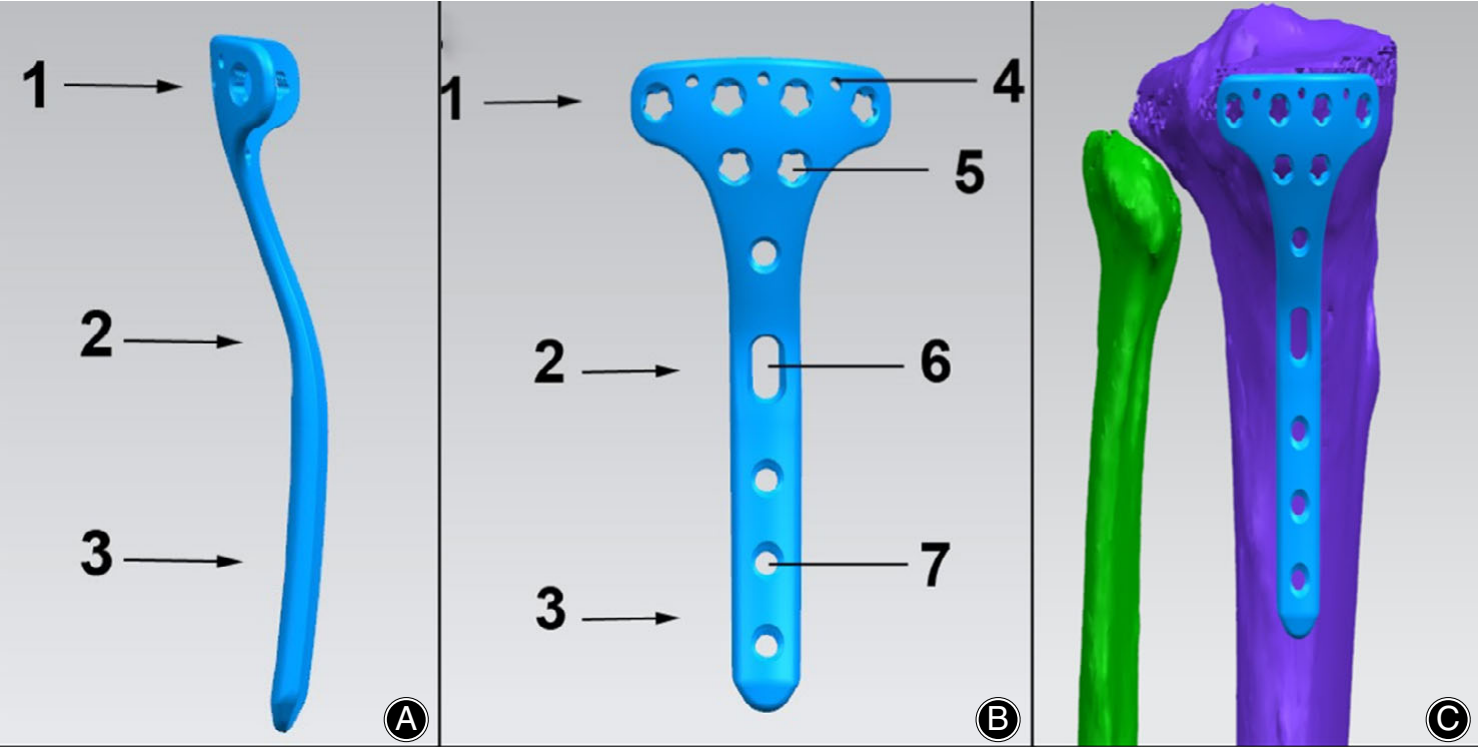
Schematic figure of the modified posteromedial anatomic locking plate. (A) lateral view. (B) Posterior view. (C) Plate used in posteromedial tibial plateau area. (1, head of plate; 2, waist of plate; 3, body of plate; 4, K‐wire hole; 5, locking screw hole in the head; 6, sliding compression hole; 7, locking screw hole in the body).

According to the structure of the posteromedial tibial plateau in a Chinese population and anatomic locking plates for the posteromedial tibial plateau fracture accessible, we made modifications to obtain a new model (Fig. 1). It was oblique “T” shaped to fit the posteromedial structure of the tibial plateau, with a wide proximal end that achieved excellent wrapping of the posteromedial condyle. It had six 30° universal locking holes in two rows to ensure precise screw insertion in the head, which fixed the plate with the posteromedial side of the tibial plateau. Four proximal row screws paralleled with the plateau could buttress powerfully, having a “raft effect.” Tiny holes in the top of the plate head were used in cases of temporary fixation or comminuted fragment fixation by K‐wire insertion and capsule sutures. It was also designed with a low profile and beveled edges. After we obtained the plate patent, the plate was made by a qualified custom‐made plate manufacturer (WASTON Medical Appliance, China). The whole structure was made of titanium. Finally, the plates obtained were completely consistent with our design in terms of their shape and distribution and the orientation of the screws. Our previous finite element study^16^ had identified the reliable stress distribution and stability.

### *Operation Results*

**Fig 2:**
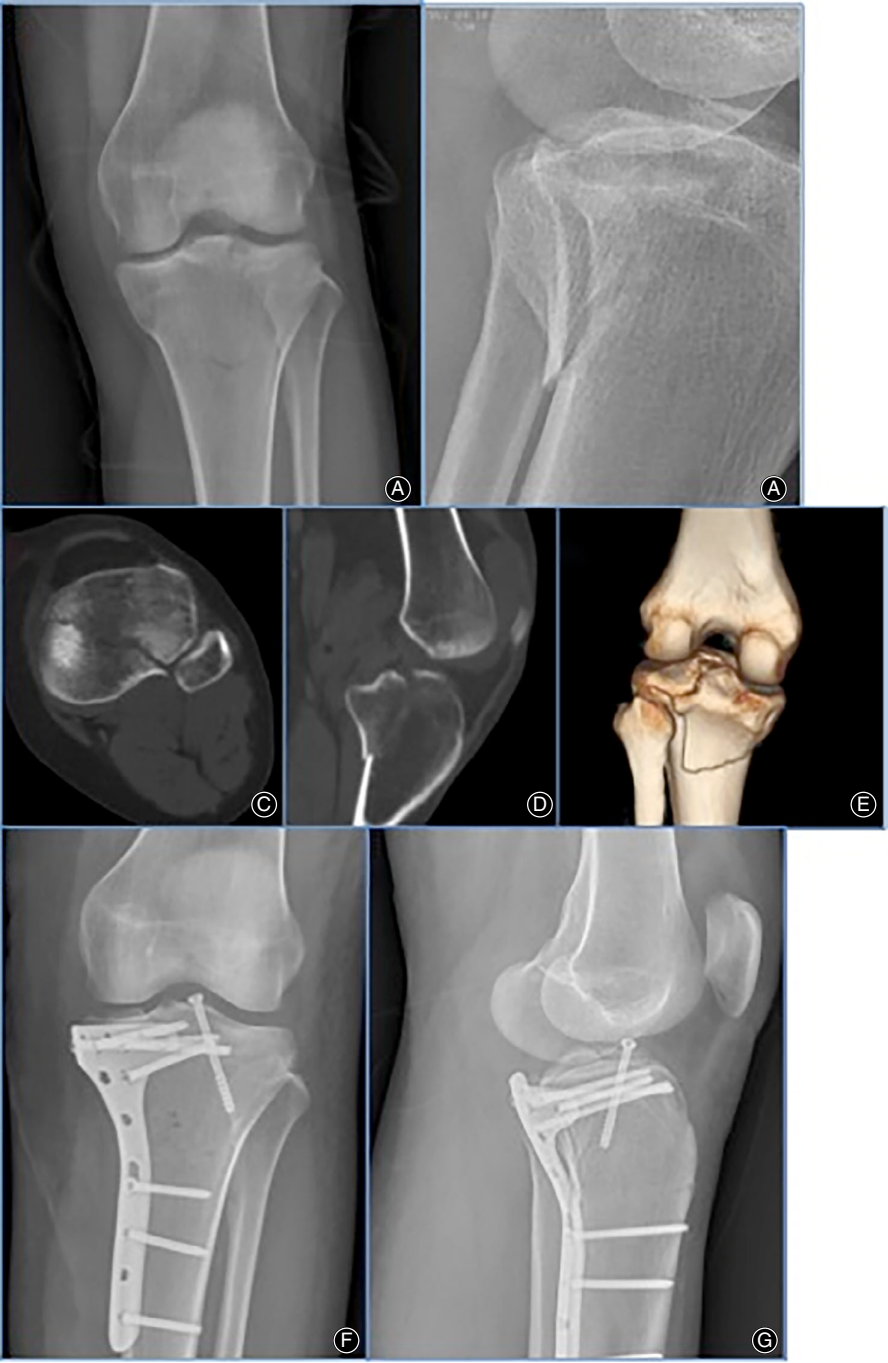
One female patient, 34 years old, with posteromedial tibial plateau fracture with anterior cruciate avulsion fracture caused by a car accident when riding a battery bike. (A, B) Preoperational anteroposterior and lateral radiographs of one posterolateral tibial plateau fracture. (C–E) More fracture details by 3‐D reconstruction of CT scan. (F, G) Anteroposterior and lateral radiographs of the patient acquired after open reduction and internal fixation with the plate.

During surgery, time and blood loss were recorded. The mean blood loss was 75 mL (range, 50–150 mL). The mean operation duration was 62 min (range, 50–90 min). Two patients’ anterior cruciate avulsion fractures were fixed with either a cable or a screw during the operation (Fig. 2). Articular surface reduction was classified as anatomic in 9 patients and acceptable in 2 patients based on a combination of direct interoperative view and fluoroscopy.

### *Clinical Improvements*

In all 11 patients, radiological fracture union was evident at 14 weeks (range, 11–18 weeks). The final follow up Tegner–Lysholm function score, the Rasmussen functional score, and the Rasmussen anatomic score were recorded (Table 1). The mean Tegner–Lysholm function score was 89.1 (range, 80 to 96). Accordingly, postoperatively, 1 patient (9.1%) achieved excellent outcomes, 9 (81.8%) good, and 1 (9.1%) fair. The mean Rasmussen functional score was 27.3 (range, 25 to 28). Nine patients (81.8%) obtained excellent functional outcomes, with two others (18.2%) obtaining good outcomes according the Rasmussen functional score. The mean Rasmussen anatomic score was 16 (range, 15 to 18), with excellent reduction quality achieved in 1 case (9.1%) and good reduction quality achieved in all others (90.9%). Symptoms of knee instability or severe pain were not found in all cases. The mean knee arc of motion was 137° (range, 122°–153°).

**TABLE 1:** Clinical findings

Case number	Blood loss (mL)	Operation duration (min)	Tegner–Lysholm score	Rasmussen functional score	Rasmussen anatomical score
1	50	70	90	28	18
2	70	55	96	28	15
3	50	60	90	27	15
4	150	90	88	28	15
5	60	70	85	25	17
6	50	50	80	27	16
7	100	80	91	26	16
8	50	80	88	28	16
9	50	60	93	27	17
10	60	70	88	28	16
11	80	70	91	28	15

### *Complications*

No evidence of knee instability was observed. No flexion contractures or extensor lag was seen. No infection, deep vein thrombosis, or graft site morbidity was observed at the follow‐up. Specifically, no case of reduction loss or internal fixation failure was reported.

## Discussion

Medial tibial plateau fractures are caused by a high‐energy mechanism of injury and account for approximately 10% of all tibial plateau fractures^3^, ^17^. In addition, there can be a transient knee dislocation that can cause tears of the cruciates and the MCL, resulting in both valgus and anteroposterior instability^5^. It is generally understood that Schatzker type IV fractures are high energy injuries leading to excess axial load combined with varus stress on the knee.

Several retrospective studies identify the posteromedial fragment in 29%–74% of all bicondylar tibial plateau fractures, an incidence that until recently was largely underappreciated if addressed at all^7^, ^18^. In morphology studies based on CT^12^, ^19^, the bone blocks of posteromedial condylar splits were found to be larger while those of posterolateral condylar were smaller. Posteromedial fractures are highly unstable injuries with a large posteromedial condyle fragment prone to posterior shearing and the risk of popliteal vessel injury. Cuellar *et al*.^7^ report that although they may initially seem nondisplaced after injury, posteromedial fragments are more likely to displace during knee range of motion exercises in non‐weight‐bearing conditions. Some authors recommend a separate fixation for this fragment to maintain knee stability^2^, ^8^.

Fractures of the anterior and lateral aspect of the tibial plateau and undisplaced posterior fragments can usually be stabilized through a standard anterolateral approach^11^, ^20^. However, reduction loss that received anterolateral and medial plate fixation in bicondylar tibial plateau fractures was reported to be as high as 48.5%^6^, ^21^. There was no plate located at the posterior aspect that could be regarded the possible cause of the high rate of reduction loss. The screw cannot rigidly fix the posteromedial fracture fragments^6^. When fixed with a lateral locking plate, the lateral screws are positioned in the coronal plane and are frequently parallel to the fracture line, which may fail to capture the posteromedial fragment^22^. In a study of factors related to reduction loss in bicondylar tibial plateau fractures, Weaver *et al*.^15^ reported that posteromedial coronal fractures were associated with a high rate of reduction loss. Operative treatment of this type of fracture requires direct exposure and posterior stabilization.

### *Approaches for Posteromedial Tibial Condyle*

Although many approaches have been reported to achieve appropriate exposure, there is no general consensus^2^, ^6^, ^8^, ^23^, ^24^. As for split fractures of the posteromedial condyle, traditionally, the anteromedial approach^23^ has been adopted to expose the medial posterior condyle. In this approach, the fracture site is shown from the lateral side; however, the medial collateral ligament (MCL) is easily injured during dissection. When dissecting over a large area, flap margin necrosis is likely to occur. Moreover, the limited exposure also adds to the difficulty of reduction and fixation. The Lobenhoffer approach^24^ allows for visualization of the posteromedial tibia and, if needed, can be applied laterally to allow access to the posterior and even posterolateral tibia. The neurovascular bundle is not exposed and, therefore, is protected within the posterior soft tissues. The visualization provided by the Lobenhoffer approach allows for accurate reduction of the extraarticular portion of the fracture followed by placement of a posterior antiglade plate. Currently, a posteromedial approach is widely applied in the treatment of posterior medial condylar fractures^8, 25^. Satisfactory results have been achieved using this incision to expose the posterior medial condylar tibial plateau fracture.

The posteromedial key fragment may displace distally and medially, especially when the knee is flexed, so a posterior‐based buttress plate or reconstruction plate internal fixation is proposed to maintain stability. Zeng *et al*.^22^ showed in a biomechanical study that a posterior‐based buttress technique was biomechanically the most stable *in vitro* fixation method for posteromedial split tibial plateau fractures when compared with AP screws and an anteromedial‐based limited contact dynamic compression plate. A posterior T‐shaped buttress plate produces significantly greater stability in controlling the subsidence of the posteromedial fragment under axial loading. Bruuner *et al*.^8^ described a posterior approach for the direct reduction and fixation of the posteromedial fragment, using a T‐shaped anti‐glide plate placed to stabilize the fragment, and all patients were highly satisfied with the postoperative result after a mean follow up of 39 months.

### *Traditional Anatomic Plate for Posteromedial Tibial Plateau*

Two kinds of anatomic T‐shaped buttress plates for the posteromedial tibial plateau are mainly used in clinic. One is a non‐locking plate that allows contouring if necessary but lacks angular stability. The direction of screws is hard to control, and they can penetrate the articular surface. The other kind is a locking plate with only three proximal screw holes in the proximal row. As shown previously, fragments of posteromedial fractures are often quite large. The proximal part may fail to wrap the entire posteromedial fragment and be difficult to contour during surgery. To better use the internal fixation of the region and to solve the problem mentioned above, we have customized a modified oblique T‐shaped buttress plate.

### *Modified Anatomic Locking Plate for Posteromedial Tibial Plateau*

The modified locking plate for the posteromedial tibial plateau is also anatomically designed. It fits the posteromedial structure of the tibial plateau and reduces the need for plate contouring during surgery, potentially leading to shorter operative duration, lower blood loss, and decreased plate failure. Our early comparative finite element study^16^ showed that it had superior performance in posterointernal tibial plateau fracture fixation and could serve as a suitable clinical alternative fixation method. The new anatomic locking plate fits anatomically, requiring no additional manipulation. In addition, the proximal part of the plate is more scientific and reasonable. It has six 30° universal locking holes to ensure precise screw insertion in the head, which fixes the plate with the posteromedial side of the tibial plateau. The wide proximal part can achieve excellent wrapping of the posteromedial condyle. Four proximal row screws paralleled with the plateau could buttress powerfully, having a “raft effect,”^26^ which provides firm strength holding the articular surface. Not only an effective buttress can be achieved, but also compression fixation of large fragments. According to the Rasmussen functional/anatomical score, all patients achieved “perfect or good” results. The plate was proven to be safe and effective in a small‐sample‐size population (11 patients) during a 19 to 60‐month follow‐up.

### *Limitations of the Study*

There are some limitations in our study. The research is based on a retrospective clinical evaluation with a relatively small sample size. A large‐sample study needs to be conducted to prove the efficacy of the fixation. In addition, further biomechanical studies are required to evaluate buttress performance, compression forces, and comparative features against traditional buttress line plates *via* the posteromedial approach. Finally, reduction loss was evaluated through simple radiographs in this study. CT is required to more accurately evaluate the existence of reduction loss, but in the present study it was impossible to perform follow‐up CT for all patients.

### *Conclusion*

In conclusion, the modified anatomic locking plate for the posteromedial tibial plateau was proven to be safe and effective in a small‐sample‐size population (11 patients) during a 19 to 60‐month follow‐up. The plate fit the irregular posteromedial tibial plateau well, without requiring additional contouring. Therefore, this new plate may be a favorable internal fixation choice for treatment of posteromedial plateau fractures.

## References

[1] Subasi M , Kapukaya A , Arslan H , Ozkul E , Cebesoy O . Outcome of open comminuted tibial plateau fractures treated using an external fixator. J Orthop Sci, 2007, 12: 347–353. 17657554 10.1007/s00776-007-1149-7

[2] Luo CF , Sun H , Zhang B , Zeng BF . Three‐column fixation for complex tibial plateau fractures. J Orthop Trauma, 2010, 24: 683–692. 20881634 10.1097/BOT.0b013e3181d436f3

[3] Chang SM , Zhang YQ , Yao MW , Du SC , Li Q , Guo Z . Schatzker type IV medial tibial plateau fractures: a computed tomography‐based morphological subclassification. Orthopedics, 2014, 37: e699–e706. 25102505 10.3928/01477447-20140728-55

[4] Morin V , Pailhe R , Sharma A , *et al* Moore I postero‐medial articular tibial fracture in alpine skiers: surgical management and return to sports activity. Injury, 2016, 47: 1282–1287. 27037028 10.1016/j.injury.2016.03.024

[5] Schatzker J , McBroom R , Bruce D . The tibial plateau fracture. The Toronto experience 1968–1975. Clin Orthop Relat Res, 1979, 138: 94–104. 445923

[6] Kim CW , Lee CR , An KC , *et al* Predictors of reduction loss in tibial plateau fracture surgery: focusing on posterior coronal fractures. Injury, 2016, 47: 1483–1487. 27178768 10.1016/j.injury.2016.04.029

[7] Cuellar VG , Martinez D , Immerman I , Oh C , Walker PS , Egol KA . A biomechanical study of posteromedial tibial plateau fracture stability: do they all require fixation?. J Orthop Trauma, 2015, 29: 325–330. 25591035 10.1097/BOT.0000000000000277

[8] Brunner A , Honigmann P , Horisberger M , Babst R . Open reduction and fixation of medial Moore type II fractures of the tibial plateau by a direct dorsal approach. Arch Orthop Trauma Surg, 2009, 129: 1233–1238. 19238408 10.1007/s00402-009-0841-9

[9] Immerman I , Bechtel C , Yildirim G , Heller Y , Walker PS , Egol KA . Stability of the posteromedial fragment in a tibial plateau fracture. J Knee Surg, 2013, 26: 117–126. 23288766 10.1055/s-0032-1319780

[10] Kumar P , Agarwal S , Kumar D , Rajnish RK , Jindal K . Rim plating for a rare variant of posteromedial tibial condyle fracture; partial coronal split, akin to Hoffa's fracture, associated with multi‐ligament injuries and central depression. Trauma Case Rep, 2019, 20: 100174. 30815530 10.1016/j.tcr.2019.100174 PMC6378844

[11] Gosling T , Schandelmaier P , Marti A , Hufner T , Partenheimer A , Krettek C . Less invasive stabilization of complex tibial plateau fractures: a biomechanical evaluation of a unilateral locked screw plate and double plating. J Orthop Trauma, 2004, 18: 546–551. 15475851 10.1097/00005131-200409000-00011

[12] Barei DP , O’Mara TJ , Taitsman LA , Dunbar RP , Nork SE . Frequency and fracture morphology of the posteromedial fragment in bicondylar tibial plateau fracture patterns. J Orthop Trauma, 2008, 22: 176–182. 18317051 10.1097/BOT.0b013e318169ef08

[13] Higgins TF , Klatt J , Bachus KN . Biomechanical analysis of bicondylar tibial plateau fixation: how does lateral locking plate fixation compare to dual plate fixation?. J Orthop Trauma, 2007, 21: 301–306. 17485994 10.1097/BOT.0b013e3180500359

[14] Yoo BJ , Beingessner DM , Barei DP . Stabilization of the posteromedial fragment in bicondylar tibial plateau fractures: a mechanical comparison of locking and nonlocking single and dual plating methods. J Trauma, 2010, 69: 148–155. 20622588 10.1097/TA.0b013e3181e17060

[15] Weaver MJ , Harris MB , Strom AC , *et al* Fracture pattern and fixation type related to loss of reduction in bicondylar tibial plateau fractures. Injury, 2012, 43: 864–869. 22169068 10.1016/j.injury.2011.10.035

[16] Fancheng C , Silin W , Zeyuan Z , *et al* Stress and stability of newly designed medial anatomic locking plates and traditional fixations in the treatment of posterointernal tibial plateau fracture: a comparative finite element study. Int J Clin Exp Med, 2018, 11: 12956–12963.

[17] Zeltser DW , Leopold SS . Classifications in brief: Schatzker classification of tibial plateau fractures. Clin Orthop Relat Res, 2013, 471: 371–374. 22744206 10.1007/s11999-012-2451-z PMC3549155

[18] Molenaars RJ , Solomon LB , Doornberg JN . Articular coronal fracture angle of posteromedial tibial plateau fragments: a computed tomography fracture mapping study. Injury, 2019, 50: 489–496. 30392718 10.1016/j.injury.2018.10.029

[19] Zhu Y , Meili S , Dong MJ , *et al* Pathoanatomy and incidence of the posterolateral fractures in bicondylar tibial plateau fractures: a clinical computed tomography‐based measurement and the associated biomechanical model simulation. Arch Orthop Trauma Surg, 2014, 134: 1369–1380. 25077782 10.1007/s00402-014-2037-1

[20] Cho JW , Kim J , Cho WT , *et al* Approaches and fixation of the posterolateral fracture fragment in tibial plateau fractures: a review with an emphasis on rim plating via modified anterolateral approach. Int Orthop, 2017, 41: 1887–1897. 28735430 10.1007/s00264-017-3563-6

[21] He QF , Sun H , Shu LY , *et al* Tibial plateau fractures in elderly people: an institutional retrospective study. J Orthop Surg Res, 2018, 13: 276. 30384857 10.1186/s13018-018-0986-8 PMC6211492

[22] Zeng ZM , Luo CF , Putnis S , Zeng BF . Biomechanical analysis of posteromedial tibial plateau split fracture fixation. Knee, 2011, 18: 51–54. 20117003 10.1016/j.knee.2010.01.006

[23] Chen HW , Chen CQ , Yi XH . Posterior tibial plateau fracture: a new treatment‐oriented classification and surgical management. Int J Clin Exp Med, 2015, 8: 472–479. 25785019 PMC4358474

[24] Hake ME , Goulet JA . Open reduction and internal fixation of the posteromedial tibial plateau via the Lobenhoffer approach. J Orthop Trauma, 2016, 30: S35–S36. 27441936 10.1097/BOT.0000000000000582

[25] Carlson DA . Posterior bicondylar tibial plateau fractures. J Orthop Trauma, 2005, 19: 73–78. 15677921 10.1097/00005131-200502000-00001

[26] Jian Z , Ao R , Zhou J , Jiang X , Zhang D , Yu B . A new anatomic locking plate for the treatment of posterolateral tibial plateau fractures. BMC Musculoskelet Disord, 2018, 19: 319. 30185201 10.1186/s12891-018-2216-2 PMC6123955

